# Genome-centric metagenomics reveals the host-driven dynamics and ecological role of CPR bacteria in an activated sludge system

**DOI:** 10.1186/s40168-023-01494-1

**Published:** 2023-03-22

**Authors:** Yulin Wang, Yulin Zhang, Yu Hu, Lei Liu, Shuang-Jiang Liu, Tong Zhang

**Affiliations:** 1 State Key Laboratory of Microbial Technology, Shandong University, Qingdao, 266000 People’s Republic of China; 2 Environmental Microbiome Engineering and Biotechnology Laboratory, The University of Hong Kong, Hong Kong, People’s Republic of China

**Keywords:** Candidate phyla radiation (CPR), Activated sludge, Striking fluctuation, Biogeochemical cycling, Bacterial evolution

## Abstract

**Background:**

Candidate phyla radiation (CPR) constitutes highly diverse bacteria with small cell sizes and are likely obligate intracellular symbionts. Given their distribution and complex associations with bacterial hosts, genetic and biological features of CPR bacteria in low-nutrient environments have received increasing attention. However, CPR bacteria in wastewater treatment systems remain poorly understood. We utilized genome-centric metagenomics to answer how CPR communities shift over 11 years and what kind of ecological roles they act in an activated sludge system.

**Results:**

We found that approximately 9% (135) of the 1,526 non-redundant bacterial and archaeal metagenome-assembled genomes were affiliated with CPR. CPR bacteria were consistently abundant with a relative abundance of up to 7.5% in the studied activated sludge system. The observed striking fluctuations in CPR community compositions and the limited metabolic and biosynthetic capabilities in CPR bacteria collectively revealed the nature that CPR dynamics may be directly determined by the available hosts. Similarity-based network analysis further confirmed the broad bacterial hosts of CPR lineages. The proteome contents of activated sludge-associated CPR had a higher similarity to those of environmental-associated CPR than to those of human-associated ones. Comparative genomic analysis observed significant enrichment of genes for oxygen stress resistance in activated sludge-associated CPR bacteria. Furthermore, genes for carbon cycling and horizontal gene transfer were extensively identified in activated sludge-associated CPR genomes.

**Conclusions:**

These findings highlight the presence of specific host interactions among CPR lineages in activated sludge systems. Despite the lack of key metabolic pathways, these small, yet abundant bacteria may have significant involvements in biogeochemical cycling and bacterial evolution in activated sludge systems.

Video Abstract

**Supplementary Information:**

The online version contains supplementary material available at 10.1186/s40168-023-01494-1.

## Introduction

Advances in metagenomic techniques have enabled a more efficient pathway to get the missing pieces in the intricate puzzle of the tree of life. The discoveries of bacterial and archaeal genomes represented previously unknown lineages have constantly broadened microbial diversity and amended the phylogenetic structure of the tree of life, pushing the common ancestor of bacteria and archaea deeper into the past [1, 2]. Among these newly reported bacterial lineages, a group of candidate phyla of mainly uncultivated bacteria that have been identified with metagenomics forms monophyletic radiation [3–5]. This radiation was defined as candidate phyla radiation (CPR, also referred to as Patescibacteria). In addition to the important role of CPR in studying bacterial and cellular evolutionary history, CPR has been inferred to represent > 15% of all bacterial diversity and contains over 70 different phyla [4]. The widely reported candidate phyla, e.g., Saccharibacteria (TM7), Parcubacteria (OD1), Gracilibacteria (BD1-5), and Microgenomates (OP11), are assigned to the CPR phylum (Patescibacteria) and placed into class-level in Genome Taxonomy Database (GTDB) [6]. These four CPR classes represent > 75% of all reported CPR members in GTDB.

While the first CPR organism (*Candidatus* Nanosynbacter lyticus strain TM7x) was co-cultivated from human oral [7], 16S rRNA gene sequence analyses have demonstrated that CPR bacteria could be found in a wide range of environments, including terrestrial [3, 4], freshwater [2, 8–10], and marine [11, 12] ecosystems. CPR bacteria predominate in groundwater, lake, and other aquifers with limited nutrients and oxygen [13, 14]. However, when considering the fact that a single copy 16S rRNA gene is typical for CPR lineages [4], the relative abundance of CPR organisms estimated using 16S rRNA gene sequences without gene copy number correction may be underestimated. Genome-resolved metagenomics provides new insights into the ecological distribution and roles of CPR organisms. He et al. [15] recovered 540 CPR bacterial genomes from groundwater metagenomes and demonstrated that CPR organisms accounted for up to ~ 40% of microbial communities of groundwater (bulk biomass onto 0.1 µm filter). Compared with the groundwater environment, the activated sludge (AS) system in wastewater treatment plants (WWTPs) is a eutrophic and aerobic engineered ecosystem with much higher biomass concentration and complex microbial diversity. Despite the documentation of CPR bacterial genomes recovered from AS [11], the microbial diversity and temporal variation pattern of CPR bacteria in AS systems are barely discussed.

A common feature of CPR organisms is their small cell sizes (200–300 nm) [7, 8] and extremely reduced genome sizes (0.85 ± 0.23 Mb). CPR bacteria often lack the complete pathways for the biosynthesis of amino acids, lipids, and nucleotides [2, 4, 15, 16]. Moreover, some CPR bacteria (e.g., *Ca*. Katanobacteria and *Ca*. Dojkabacteria (WS6)) cannot even de novo synthesize cell envelopes due to incomplete lipids and/or peptidoglycan synthesis [13]. Despite the reduced metabolic platforms of CPR organisms, analyses of gene repertoires and metabolic capacities revealed the highly divergent genome content among CPR lineages, even within the same lineage. Moreover, the divergence between CPR genome content showed complex relationships with correlated microbial community members and environmental types. For example, the comparative genomic analysis revealed that animal-associated Saccharibacteria have smaller gene repertoires than their environmental counterparts [9]. In contrast to the numerous genes that cope with oxidative stress in soil-associated CPR organisms, genomes recovered from the anoxic groundwater environment lack genes relate to oxygen metabolism [9, 16]. While these genetic comparisons between CPR lineages from different environments provide important clues to exploring their niche adaptation strategies, it is still poorly understood how CPR bacteria survive and interact with other organisms in AS.

Despite a generally limited biosynthetic potential, these ubiquitous CPR bacteria probably contribute to biogeochemical cycling [2, 17–19]. Many members of CPR have been reported to encode genes involved in lactate, formate, and/or ethanol production. Several CPR genomes recovered from groundwater encode copper nitrite reductase (*nirK*) and/or an NADPH nitrite reductase (*nirB*). The co-culture experiments have confirmed the obligate symbiotic lifestyle of CPR organisms. In addition to getting essential compounds from its hosts, one epiparasitic bacterium from the Saccharibacteria had been reported to act as a bacteriophage and lyse foaming bacteria in WWTPs [20]. Given the roles of CPR organisms in biogeochemical cycling and microbial interactions, it is of particular interest to explore what ecological role they play in wastewater treatment systems.

In this study, we took advantage of our previously reported nine-year time-series AS metagenomes (97 samples) from Shatin (ST) WWTP in Hong Kong, China [21], and sequenced 22, 13, and 13 newly collected AS samples taken monthly from ST, Shek Wu Hui (SWH), and Stanley (STL) WWTPs, respectively. Using these AS metagenomes, we recovered non-redundant bacterial and archaeal metagenome-assembled genomes (MAGs), including 135, 31, and 28 CPR bacterial MAGs from ST, SWH, and STL WWTPs, respectively. The long-term time-series data of ST AS enabled the characterization of the temporal dynamics of CPR communities and the inference of their putative hosts. Comparative genomic analysis among CPR from different environments was performed to predict the origin of abundant CPR in WWTPs. The roles of CPR organisms in the carbon cycling and microbial evolution process were further discussed based on the genome-resolved analyses. Overall, these results advanced our understanding of the ecological roles of CPR bacteria in bioengineered systems that have not yet been fully resolved.

## Methods

### Sample collection, DNA extraction, and metagenomic sequencing

Previously reported AS metagenomes [21, 22] and newly collected AS samples were integrated and used to recover as many CPR genomes as possible. AS samples used in the present study were collected from three biological WWTPs, i.e., Shatin (ST) WWTP, Shek Wu Hui (SWH) WWTP, and Stanley (STL) WWTP. Three batches of monthly sampling campaigns were performed from June 2007 to December 2015 (ST WWTP) [21], from April 2017 to December 2017 (ST WWTP), and from January 2018 to January 2019 (ST, SWH, and STL WWTPs) [22], resulting 119, 13 and 13 AS samples taken from ST, SWH, and STL WWTPs, respectively. This work provided a long-term temporal metagenomic study for AS ecosystem, which is of great importance for microbial network inference. Other detailed information in terms of wastewater treatment performance and operational parameters could be found in our previous works [21, 22]. In addition to the AS samples, we also collected two sets of effluent samples in March 2018 and April 2018 from ST, SWH, and STL WWTPs to evaluate the distribution of CPR organisms in the effluent after secondary sedimentation tanks.

As for the newly collected AS samples, 1 mL aliquot of each diluted sample was centrifuged to obtain a pellet of ~ 200 mg, which was subjected to DNA extraction with the FastDNA Spin Kit for Soil (MP Biomedicals, OH, USA). The extracted DNA samples were then sequenced on an Illumina X Ten (150 bp paired-end reads, 350 bp insert size) at Beijing Novogene Bioinformatics Technology Co., Ltd. (Beijing, China), generating a total of 46 new metagenome datasets with sequencing amounts of 12.2 ± 1.2 Gb (average ± standard deviation).

### CPR bacterial MAGs recovery

Metagenomic reads were quality controlled using the “read_qc” module of MetaWRAP (v1.3) [23]. AS metagenomes generated from the years 2017 and 2018 co-assembled using MEGAHIT (–min-contig-len 800 and –presets meta-large) (v1.1.1) [24]. The co-assembly results of each year from ST, SWH, and STL WWTPs were respectively imported to MetaWRAP to recover bacterial and archaeal MAGs using metabat2 (v2.9.1) [25], maxbin2 (v2.2.4) [26], and concoct (v0.4.0) [27]. The initial recovered MAGs were consolidated using the “bin_refinement” module of MetaWRAP. The completeness and contamination of the newly recovered MAGs were estimated using CheckM (v1.0.18) [28] with lineage-specific workflow and default parameters. The recovered ST MAGs from the years 2017 and 2018 were integrated with the previously published 920 MAGs [21] for dereplication using dRep (v2.3.2) [29] at the thresholds of 90% Mash similarity for the primary clustering and 99% average nucleotide identity (ANI) for the secondary clustering. Only MAGs with completeness ≥ 50% and contamination ≤ 10% were retained for downstream analysis. Taxonomy assignment was performed using GTDB-Tk (v1.5.1) [30]. Given the reported CPR bacteria often missing many “universal” bacterial markers, the genome qualities of bacterial MAGs assigned to the phylum Patescibacteria were reevaluated using a set of markers (43 genes) specifically for CPR lineages (Supplementary Table S1).

### Phylogenetic tree construction

The “universal” marker genes of the newly recovered CPR MAGs from AS metagenome and the selected reference genomes represented the CPR lineages were classified using the “identity” module of GTDB-Tk (v1.5.1) [30]. The identified marker genes were then aligned and concatenated using the “align” module of GTDB-Tk [30]. FastTree (v2.1.10) [31] was then used to infer a genome tree based on the concatenated alignment of the identified markers genes under the WAG + GAMMA model [32]. The genome tree was imported into iTOL [33] for further refinements.

### Microbial community dynamics and network analysis

In the present study, we focused on the microbial dynamics in ST WWTP, because the long-term sampling campaign of that WWTP could provide reliable community-wide and population-resolved traits longitudinally, which will also enable us to infer a robust microbial network. The relative abundance of the dereplicated ST MAGs was calculated using CoverM (v0.2.0, https://github.com/wwood/CoverMs), which mapped metagenomic sequences to the MAGs with the cutoff of read identity 70% and minimum read aligned percentage 50%. Microbial networks were inferred using extended local similarity analysis (eLSA, v1.0.6). The *P* value was calculated with theoretical approximation (-p theo). The microbial network inference was based on the whole microbial community dynamic profile. The strong (local similarity (LS) score ≥ 0.8 or ≤  − 0.8) and statistically significant (*P* value ≤ 0.05; false-discovery rate ≤ 0.01) correlations were retained from the eLSA analysis results. Microbial networks were visualized using Cytoscape (v3.7.1) [34]. The taxon nodes in the network were clustered using Markov CLustering Algorithm (MCL) in the Cytoscape plugin clusterMaker [35] based on the values of the local similarity score.

To examine the inferred CRP host associations, we simplified the AS microbial communities through a long-term 100 ppm penicillin treatment (60 days). Briefly, the influent of Shatin WWTP was filtered with 0.22-μm membranes (Advantec MFS, USA), and penicillin was added to the filtrate with a final concentration of 100 ppm to prepare the enrichment media. Fifty-milliliter AS sample from ST WWTP was centrifuged at 4,500 rpm for 15 min to discard the supernatant. Pellets were collected and resuspended with 50 mL enrichment solution in a 100 mL conical flask. The conical flask was placed in a shaker at 180 rpm and incubated at room temperature for 2 months of successive transfer of biomass every 7 days with new enrichment media. The DNA of antibiotic-treated AS was extracted and used for metagenomic sequencing and genome binning. FastANI [36] was used to link the relationships between the microbes selectively enriched by penicillin and the microbes in AS used for network inferences at an ANI cutoff of 99%. We then examined whether the putative hosts of the given CPR bacterium inferred by network analysis were selectively enriched in antibiotic (penicillin) treated AS community.

### Genome annotation

The open reading frames (ORFs) for the MAGs were predicted using Prodigal (v2.6.3) [37] and subsequently annotated by comparing predicted ORFs to the KEGG [38], NCBI nr, and EggNOG databases using GhostKOALA (v2.2) [39], Diamond (v0.9.22.123) [40], and EggNOG-mapper (v2.15) [41], respectively. The completeness of metabolic pathways of CPR MAGs was estimated using EnrichM (v0.5.0; https://github.com/geronimp/enrichM). Key metabolic pathways (e.g., amino acids and vitamins) were manually checked using KEGG Mapper (v4.1) [42] based on the KEGG orthology (KO) assignment of predicted ORFs. The carbohydrate-active enzymes (CAZy) in CPR MAGs were identified by comparing predicted ORFs to dbCAN HMMs V7 [43] using HMMSCAN [44] and subsequently summarized using an online script (https://github.com/yuboer/genome-centric-portrait-ofcellulose-hydrolysis).

### Comparative genomic analyses

To conduct comparative genomic analysis between CPR bacteria in WWTPs and other environments, we collected publicly accessible genomes of Saccharibacteria, which is the predominant CPR lineage in WWTPs, from NCBI, PATRIC, ggkbase, and IMG [9]. These saccharibacterial genomes were recovered from wastewater treatment systems, groundwater, seawater, lake, hydrothermal soil, permafrost, and human (oral and gut). Genomes collected from the public databases were combined with the newly recovered CPR MAGs from ST, SWH, and STL WWTPs in the present study for the downstream comparative genomic analyses. The pangenome of investigated CPR organisms was created using anvi’o, following the standard pangenome workflow [45]. Briefly, amino acid similarities between the genes predicted from studied CPR genomes were calculated using BLAST (blastp, v2.12.0) [46]. Protein families were clustered using MCL [47] with an inflation parameter set to 10 based on the all-against-all gene similarity matrix. We then investigated the proteome content similarities between the studied CPR genomes using principal-coordinate analysis (PCoA). The distance between different genomes was calculated based on the matrix of presence/absence protein families with 5 or more member sequences [9]. This presence/absence matrix was visualized using pheatmap library in R (v4.0.3) [48]. The protein families displayed in the heatmap were hierarchically clustered based on Euclidean distance.

We next compared the difference among CPR organisms from different environmental categories in both metabolic pathways and protein families. The obtained KO frequency matrix of annotated MAGs was used to perform statistical tests (EnrichM, v0.5.0) to identify the significantly enriched KEGG steps (i.e., reactions) involved in metabolic modules between any two environmental categories associated CPR organisms. The Fisher exact statistic test was used to identify the differentially distributed protein families (false-discovery rate-corrected value of *P* < 0.05) between CPR organisms from different environmental categories based on the presence/absence matrix. For the annotation of each clustered protein family, the most common KEGG annotation among its member sequences was selected [9].

### Horizontal gene transfer in CPR organisms

In this study, we investigated the horizontal gene transfer occurring between CPR and other prokaryotic (bacterial or archaeal) organisms and phages in ST WWTP. The non-redundant MAGs recovered from ST metagenomes were used to look for gene transfer events. Gene sequences of each CPR genome were searched against other bacterial and archaeal genomes using Blast (blastn, v2.12.0) [46]. We retained the blast hits with > 99% identity and that are larger than 500 bp [49]. To reduce the false positives generated from the wrongly binned contigs (e.g., contigs that are simultaneously in different MAGs), we removed the blast hits that involved query or reference coverage > 80%. Moreover, gene sequences of each CPR genome were searched against a prokaryotic virus catalog from WWTPs in Hong Kong[50]. ORFs in the horizontally transferred fragments were extracted and annotated with EggNOG-mapper (v2.15) [41].

## Results

### Diverse CPR in wastewater treatment systems

**Fig. 1:**
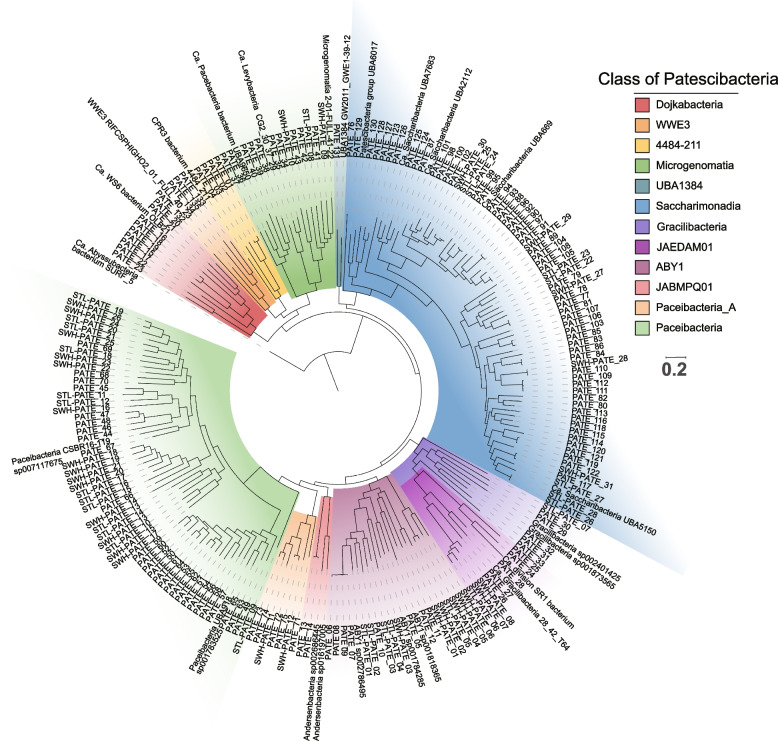
Phylogenetic placement of CPR MAGs recovered from Shatin (ST), Shek Wu Hui (SWH), and Stanley (STL) WWTPs. The phylogenetic tree was inferred based on the concatenated gene alignments of conserved genes from 194 WWTP-associated and 24 reference CPR genomes using the GTDB-Tk [30].

Genome-resolved metagenomics revealed high bacterial diversity of CPR organisms in WWTPs. We retrieved 135 CPR bacterial genomes from ST AS metagenomes. Four circular CPR genomes (i.e., PATE (Patescibacteria)_25, _28, _56, and _101) obtained in our previous work focusing on the recovery of high-quality MAGs using a hybrid assembly of Illumina short reads, and Nanopore long reads [51] have been included. In contrast, only 31 and 28 CPR bacterial genomes were respectively retrieved from SWH and STL WWTPs due to the limited number of metagenomic datasets. The size of these CPR MAGs was 0.86 ± 0.21 Mb (average ± standard deviation). The genome completeness of these PATE MAGs was 64.5 ± 7.67% (average ± standard deviation) based on the 104 universal bacterial markers identified in CheckM, which was in line with the previous low genome completeness of CPR organisms. The average completeness of these CPR MAGs significantly increased to 80.7% (Supplementary Table S2 and Figure S1) if using 43 markers specific for CPR lineages [4]. While PATE_25 is a circular genome, the completeness of PATE_25 is 95.3% even using the CPR-specific markers, suggesting the gene content of CPR lineages might be rather diverse. The pair-wise ANI comparison showed that only 0.5% of pairs shared ANI > 70% (Supplementary Table S3), which was consistent with the observed long branch length among different PATE MAGs in the phylogenetic tree (Fig. 1).

Most of the CPR organisms identified in ST WWTP were affiliated with Saccharimonadia (55), Paceibacteria (32), Microgenomatia (10), Dojkabacteria (9), and ABY1 (8) (Supplementary Table S2). This taxonomic distribution was also confirmed in the geographically different SWH and STL WWTPs. Based on the result of the taxonomic assignment (Supplementary Table S2), it is worth noting that all these PATE organisms cannot be assigned at the species level and 117 PATE MAGs might come from novel genera. A total of 68 saccharimonadial bacterial MAGs were recovered from the present study, increasing the number of deposited saccharimonadial genomes in GTDB by 12.7%.

### Striking fluctuations in the CPR community over time

**Fig. 2:**
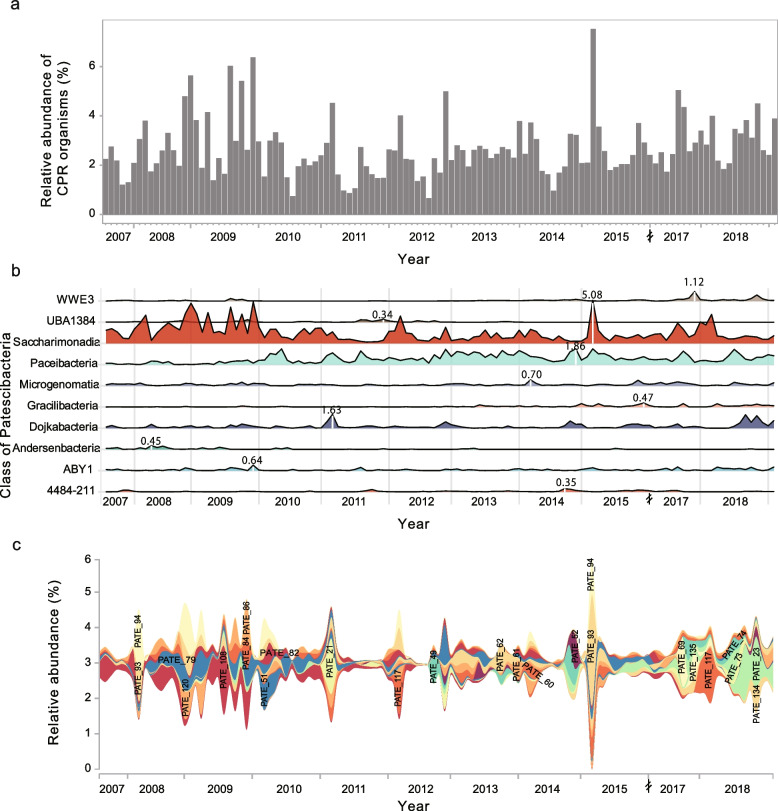
Dynamics of CPR communities in activated sludge of Shatin WWTP. **a** Relative abundance of the CPR guild in Shatin AS samples over eleven years based on the genome-wide coverage. **b** Temporal dynamics show the changes in CPR communities at the class level. **c** Temporal dynamics show the changes in CPR communities (MAGs-based), only CPR bacteria that are found in at least one AS sample with relative abundance > 0.5% are shown in this figure

This study provided a long-term temporal profile (~ 11 years) of the CPR community in the studied WWTP. The relative abundances of AS microbial community members were calculated using the genome-wide coverages. CPR guild was identified as one of the dominant bacterial populations in ST WWTP with a relative abundance of up to 7.5%, while the overall relative abundance of CPR guild varied greatly across AS samples taken from different months (Fig. 2a). Moreover, the abundance changes among different years also varied dramatically, ranging from 1.9 (2013) to 7.5 (2012). Interestingly, relatively higher abundances of CPR bacteria were generally found in winter.

In ST WWTP, the most abundant CPR lineage is Saccharimonadia with average and maximum relative abundances of 1.26 and 5.08%, respectively (Fig. 2b). While the class Paceibacteria was rare in the first 3 years (2007–2009) with an average relative abundance of 0.19%, the relative abundance of this lineage abruptly increased from 0.53% at the end of 2009 to 1.3% in February 2010 and was temporally stable in the following 8 years. In contrast, the relative abundance of Saccharimonadia substantially decreased after the end of 2009. Although the other CPR lineages (e.g., Microgenomatia, Gracilibacteria, and Dojkabacteria) transiently showed a relative abundance > 0.5% in several months, they were typically found in low abundances over years (Fig. 2b).

In addition to the dynamics of CPR at high taxonomic levels, few of them were found to be persistently dominant organisms in the studied ST WWTP (Fig. 2c). A total of 23 CPR bacteria were found in at least one AS sample with relative abundance > 0.5%. These abundant organisms displayed a seasonal dynamic pattern. Most of the CPR organisms were transient residents and replaced each other over time. Only several of the abundant CPR organisms displayed temporal synchrony (e.g., PATE_93 and _94; PATE_84 and _86). The synchronously changed CPR organisms shared close phylogenetic relationships and formed a monophyletic clade in the phylogenetic tree (Supplementary Figure S2). For example, PATE_93 and PATE_94 (98.5% ANI) were assigned to an unclassified species within the genus *Saccharimonas*. In contrast, CPR bacteria from different genera or families displayed divergent dynamic patterns (Supplementary Figure S2 and Table S4).

### Symbiotic lifestyles of CPR bacteria in AS system

**Fig. 3:**
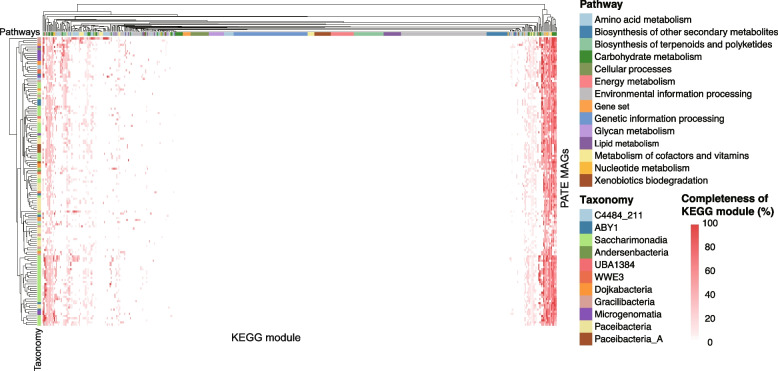
Metabolic repertoire of the newly recovered CPR MAGs. The heatmap shows the completeness of the Kyoto Encyclopedia of Genes and Genomes (KEGG) modules of the WWTP-associated CPR MAGs, arranged based on the patterns of metabolic modules’ completeness. The categories of KEGG metabolic modules and taxonomic affiliation are annotated on the top row and left column of the heatmap, respectively

The metabolic repertoire of the studied CPR genomes in AS system revealed their limited metabolic and biosynthetic capabilities. Consistent with the previously reported works, these newly recovered CPR MAGs lacked essential central energy metabolisms and pathways for most amino acids and vitamins biosynthesis (Fig. 3). Despite nor of the studied CPR MAGs possessing complete pathways for carbohydrate metabolisms, genes coding for enzymes for the core module of glycolysis (three-carbon compounds) could be widely identified in MAGs of Saccharimonadia, ABY1, Microgenomatia, and Paceibacteria. The pentose phosphate pathway was incomplete in 96.3% of the CPR MAGs. Though the core genes for glycolysis were typically absent in most MAGs of Gracilibacteria, three of them (PATE_29, _30, and _32) possessed a complete pentose phosphate pathway (Supplementary Table S5). Almost all the studied CPR bacteria did not contain all essential components to synthesize nucleotides, except for several MAGs that had complete pathways for biosynthesis of guanine and adenine ribonucleotides (e.g., PATE_29, _30, and _32 within Gracilibacteria). Besides, none of the studied CPR bacteria encoded genes for the components necessary to synthesize membrane lipids. The reduced genome size and missing of numerous metabolic and biosynthetic capacities confirmed that CPR organisms in AS system adopted symbiotic lifestyles.

### Broad bacterial host ranges inferred by time series data

**Fig. 4:**
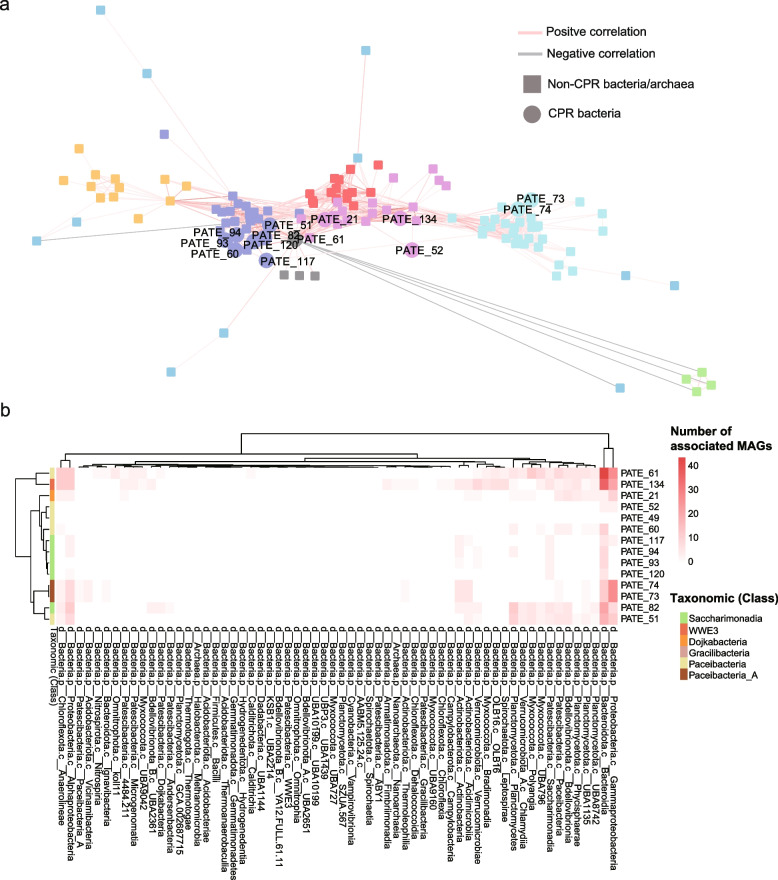
Potential hosts of CPR bacteria. **a** Similarity-based sub-network shows associations between non-CPR bacteria/archaea and the dominant CPR bacteria that are found in at least one AS sample with relative abundance > 0.5%. The edge colors show positive (red) and negative associations (blue). The node shapes represent the non-CPR bacteria/archaea (square) and CPR bacteria (circle). **b** Heatmap summarizes the taxonomic affiliation of the potential hosts of abundant CPR bacteria at the class level. The color represents the number of observed associations between the given CPR bacterium and bacterial host lineage (class)

Similarity-based microbial association network was built to capture links between microorganisms in ST WWTP and was therefore used to infer the bacteria that may serve as the host of CPR organisms. As shown in the subnetwork extracted from the entire association networks (Fig. 4a), the abundant CPR bacteria and their associated bacteria together formed three clusters based on local similarity scores. As expected, the CPR bacteria within the same cluster generally showed close phylogenetic relationships. For example, PATE_82, _93, _94, _117, and _120 that were affiliated with the same order Saccharimonadales clustered in the same module, while the organisms with distinct taxonomic affiliations (e.g., PATE_73 and PATE_74 within the family Moranbacterales of Patescibacteria) formed a different module in the network (Fig. 4a). Furthermore, the specific dynamics of the dominant CPR bacteria from the same network module (e.g., PATE_82, _93, _94, and _120) bloom with their associated abundant bacteria in winter (Supplementary Figure S3). These cohesive and seasonal dynamics suggested that the relatively higher abundances of CPR in winter might be determined by its host.

Given the topological properties of microbial network, we further accessed the taxonomic distribution of the potential CPR-associated bacteria in the entire network. We found that hosts of the CPR bacteria from the same class displayed a similar taxonomic distribution pattern. As shown in Fig. 4b, the potential hosts of the abundant Saccharimonadia were primarily identified from bacterial classes of Bacteroidia (GTDB taxonomy), Alphaproteobacteria, and Actinobacteria. BACT_151 and PROT_168 that were affiliated with Bacteroidota and Proteobacteria, respectively, were found to be common hosts of the abundant Saccharimonadia. However, these two common hosts could not be represented by any cultivated bacteria and were assigned to placeholder genera (BACT_151, g__UBA5535; PROT_168, g__QY30). In addition to the potential hosts within Bacteroidia and Alphaproteobacteria, CPR bacteria within the class Paceibacteria_A (GTDB taxonomy) showed intensive associations with bacteria within Gammaproteobacteria. In contrast to the relatively high host-specificity of Saccharimonadia (except PATE_51 PATE_61) and Paceibacteria_A, the potential hosts of WWE3, Dojkabacteria, and Paceibacteria showed more associations (links in the network) and broader taxonomic distribution, which further evidenced the broad bacterial host ranges of CPR organisms.

The 60 days of penicillin treatment further simplified the microbial community composition in the AS taken from ST WWTP. Of the 74 recovered bacterial MAGs from the penicillin treated AS, one CPR bacteria (PATE_102) that belong to the genus UBA2112 of Saccharimonadia was significantly enriched as one of the top 3 dominant organisms. The genome-wide relative abundance of PATE_102 dramatically increased from 0.006 to 4.4% (Supplementary Table S6). It is noteworthy that one (BACT_52) of the potential hosts inferred by microbial network analysis was found to be the most abundant organisms in the antibiotic-treated AS sample, demonstrating some degree of accuracy of the similarity-based network analysis for CPR host inference. Genes encoding beta-lactamase identified in both PATE_102 and BACT_52 further explained their high relative abundances after being treated with penicillin, which is a member of β-lactam antibiotics.

### Protein family analysis shows the environmental origin of CPR in AS systems

**Fig. 5:**
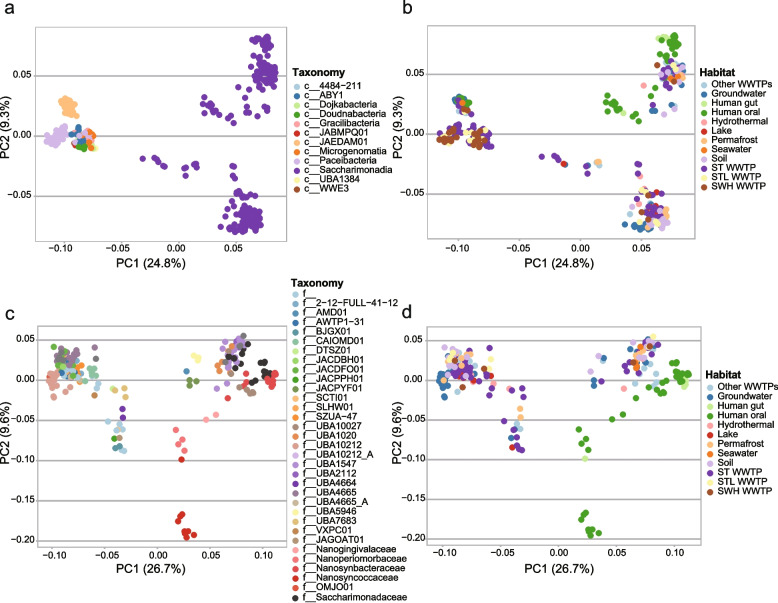
Proteome content similarity among CPR bacteria from different habitat categories. Overall proteome content similarity among all identified CPR MAGs from the studied WWTPs and public Saccharimonadia are colored based on taxonomic affiliations (**a**) and habitats (**b**). Overall proteome content similarity comparisons within the class of Saccharimonadia are shown in **c** and **d**. PCoA was computed based on the presence/absence profiles of all protein clusters with 5 or more member sequences

To examine the habitat origin of CPR in AS systems, we compared the similarities of proteome content among CPR bacteria from different habitats. We collected 202 publicly available CPR high-quality MAGs (Supplementary Table S7) from environmental (groundwater, hydrothermal, lake, permafrost, seawater, soil, and other WWTPs) and animal (human gut and oral) associated habitats, and 194 CPR MAGs recovered in the present study. Pangenomic analysis yielded 141,061 protein families. This pangenome can be considered as “open” as nearly 260 new protein families are continuously added for each additional genome considered. If we focus on the protein families with 5 or more members, the pangenome is “close” since no new protein families are added for each additional CPR MAG considered (Supplementary Figure S4). PCoA analysis of the presence/absence profile for protein families with 5 or more members [9] among CPR bacteria (Fig. 5a) revealed distinct proteome content patterns between Saccharimonadia and other CPR lineages. In addition to Saccharimonadia, JAEDAM01 and Paceibacteria on the PC2 axis (9.3% variance explained) displayed strong clustering and formed three clusters, while CPR bacteria from other CPR lineages could not be well distinguished and clustered together. There are no strong correlations between habitat types and proteome content within in given CPR class, except for human-associated Saccharimonadia that formed clusters divergent from the environmental ones (Fig. 5b).

We further examine the dissimilarities of proteome contents among saccharimonadial bacteria when considering the dominant role of Saccharimonadia in the studied AS system (Fig. 5c, d). A distinct proteome content cluster was observed from the families Saccharimonadaceae and UBA10027, which included both environmental and human-associated bacteria. Notably, the PCoA result suggested that the Saccharimonadaceae in AS may not be of human origin as proteome content in WWTPs associated MAGs were distinct from human associate ones (Fig. 5d). In contrast, the CPR MAGs clustered with other environmental CPR MAGs obtained from soil, lake, and groundwater. Despite that the studied WWTP was designed to provide service to the nearby residents, these findings indicate that the observed CPR bacteria in AS systems may originate from environmental habitats rather than be introduced by human waste in sewage.

### Proteome content differences associated with CPR niche differentiation

**Fig. 6:**
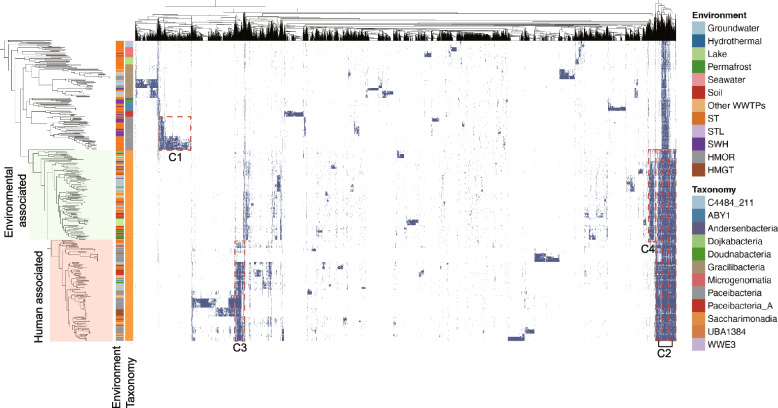
Distribution of protein families among CPR bacteria from different habitats. The heatmap shows the presence (blue) and absence (white) of protein families with 5 or more members. Protein families (columns) are hierarchically clustered based on Euclidean distance. CPR bacteria (rows) are arranged based on the phylogenomic tree. Taxonomic affiliation and environmental habitats distribution are aligned to the left of the heatmap

To figure out why AS systems selected the environmental CPR bacteria, we further examined whether there are proteome differences between human-associated and environmental CPR bacteria. The hierarchically clustered presence/absence array of protein families (with 5 or more members) in CPR bacteria revealed that multiple clusters of protein families were conserved in given CPR lineages. As shown in Fig. 6, one cluster was found to be conserved across all CPR lineages. However, this conserved cluster only contained approximately 5.1% of the protein families, indicating the high divergence of proteome content in CPR bacteria. Consistent with the previously reported work [9], these conserved protein families are related to essential cellular functions. Numerous large and small protein family clusters specific to given CPR lineages were observed. One large cluster (C1) with 468 protein families was conserved in Paceibacteria. A few small clusters were conserved in Gracilibacteria and other low abundant CPR bacteria with narrow phylogenetic distributions, which may be due to limited newly recovered MAGs and reference genomes for pangenomic analysis.

As the largest guild in WWTPs and human-associated samples, Saccharimonadia encoded one cluster (C2) that was exclusive to the other studied CPR lineages. Within Saccharimonadia, most environmental bacteria (67%) were phylogenetically clustered as a basal group (colored green) in the phylogenomic tree (Fig. 6). All the Saccharimonadia from human oral and gut by contrast clustered with several environmental associated bacteria and formed a deep-branching clade. Two clusters, C3 (107 protein families) and C4 (94 protein families), were found conserved in human-associated and environmental clades, respectively. These conserved clusters contained protein families annotated as components of organic substrates metabolism/transportations, amino acids transportations, and adaptive response to aerobic conditions.

Statistical comparison between ST WWTP and human-associated Saccharimonadia revealed that L-lactate dehydrogenase, alpha-amylase, and alpha-glucosidase were significantly enriched in human-associated Saccharimonadia. Genes encoding components of the polar amino acid transport system (e.g., substrate-binding protein, permease protein, and ATP-binding protein) were also enriched in human-associated Saccharimonadia. Additionally, CRISPR-associated proteins (cas1, cas2, and cas9) were exclusively identified in human-associated Saccharimonadia. While components involved in the ability to resist oxidative stress (e.g., superoxide dismutase (Cu–Zn, nickel, Fe–Mn families)) were significantly enriched in the WWTP-associated ones. It is noteworthy that several saccharimonadial bacteria encoded tetracycline resistance protein (*tetA*, in 9 MAGs). Besides, multidrug resistance genes, such as multidrug efflux pump (*LfrA*) and methylenomycin A resistance protein (*mmr*) were found in 7 and 12 saccharimonadial bacteria, respectively, which were not identified in any human-associated Saccharibacteria. Of these bacteria carrying antibiotic resistance genes (ARGs), PATE_82, PATE_84, and PATE_86 were observed as dominant organisms with relative abundances > 0.5% in multiple AS samples.

Comparison of overall metabolic pathways between human-associated and environmental Saccharimonadia only yielded limited pathways (partial) that were significantly enriched in a given microbial guild. Glycogen degradations (glycogen to glucose-6P) and polar amino acid transport system were enriched in human-associated Saccharimonadia. In contrast, several steps of Cytochrome o ubiquinol oxidase and Cytochrome bd ubiquinol oxidase were significantly enriched in WWTP-associated ones. These distinctions were further evident in the adaption of Saccharimonadia for the aerobic condition in AS systems.

### CPR bacteria contribute to the carbon cycle and microbial evolution process in AS system

In addition to the aforementioned genes coding components for glycolysis and pentose phosphate pathways, we also observed the potentials of fermentation in the studied AS CPR bacteria. However, only limited CPR bacteria in the studied AS systems were predicted to produce lactate (6), acetate (6), and/or formate (3) via fermentation of pyruvate. These fermenters were affiliated with specific lineages, including Dojkabacteria, Gracilibacteria, Microgenomatia, and Saccharimonadia. Of the 135 CPR bacteria recovered from ST WWTP, 80 and 111 CPR MAGs encoded genes for polysaccharide lyase (PL) and glycoside hydrolase (GH) families (Supplementary Table S8). Among the observed 28 GH families, GH1, GH5, and GH74 were frequently observed in 36.3%, 46.7%, and 24.4% of studied CPR bacteria in ST WWTP. While GH74 displayed a relatively low prevalence, genes coding for this protein are generally in multiple copies in CPR bacteria, including five abundant ones within the classes Patescibacteria (PATE_73) and Saccharimonadia (PATE_79, _82, _93, and _94).

It is predicted that horizontal gene transfer events may occur between CPR bacteria and their hosts. In the present study, we observed 84 horizontal gene transfer events from 43 CPR MAGs (Supplementary Table S9). The lengths of these transferred DNA fragments ranged from 518 to 5,730 bp. A total of 149 ORFs were predicted from these DNA fragments, while only 47.0% of them could be annotated as known COG categories (Supplementary Table S10). The transferred genes mainly belong to the categories of replication, recombination and repair and transition, ribosomal structure, and biogenesis (Supplementary Figure S5). Also, several CPR genes encoding for pilB, pilT, pilC, and secA which are involved in intracellular trafficking and secretion were predicted to be potential acquisition via horizontal gene transfer from other bacteria.

Potential CPR-phage genetic interactions were evidenced by the 24 horizontal gene transfer events from 14 CPR MAGs (Supplementary Table S11). Only 27.7% of the ORFs predicted (83) from these DNA fragments could be annotated as known COG categories (Supplementary Table S12). Although most of the transferred genes are hypothetical proteins, 30.4% of the functionally annotated genes belong to the categories of replication, recombination, and repair. Putative phage genes encode phage tail tape measure protein, phage tail sheath protein, phage portal protein, phage terminase, and Rhs family were found to be integrated with CPR genomic sequence via horizontal gene transfer from phages.

Given the small cell size of CPR bacteria, we hypothesized that these bacteria might not be efficiently filtered by conventional secondary sedimentation facilities. The relative abundances of CPR bacteria in two effluent metagenomes were approximately three to four-fold higher than that in the corresponding AS metagenomes when normalized the sequencing depth (Supplementary Figure S6), indicating that the relative abundance of CPR bacteria was moderately enriched in effluent and would likely be discharged to receiving ecosystems.

## Discussion

When considering the wide distribution of CPR across human-associated and environmental ecosystems, it is of critical importance to answer how the CPR communities changed and what kind of ecological roles they might play in a given environment. Our long-term longitudinal metagenomic analyses enabled the high-resolution characterization of changes in CPR communities over time. It is well accepted that CPR bacteria predominate in groundwater [16, 52] and lakes [14]. The temporal dynamic profile of CPR bacteria in AS system demonstrated that CPR bacteria could be particularly abundant in engineered systems under eutrophic and high dissolved oxygen conditions. A previously reported study focusing on the recovery of high-quality MAGs from AS metagenomes also reported the high relative abundance of CPR organisms in AS at a spatial scale (relative abundance of > 7%) [53]. With the newly recovered CPR MAGs, we demonstrated that AS system harbored high CPR bacterial diversity with a large proportion of novel bacteria lineages. Saccharimonadia and Paceibacteria were the most dominant CPR taxa in the studied AS system, while the routinely detected lineages in groundwater, lake, and other aquifer environments (e.g., Parcubacteria and Microgenomatia) [54, 55] were rarely identified as abundant populations in AS systems.

More importantly, the time-series metagenomic analysis in this study provided a long-term temporal profile (> 10 years) charting how CPR communities change over time. The striking fluctuations observed with respect to the high taxonomic and MAGs levels both suggested the absence of generalists for CPR bacteria in AS system. As reported in our previous study, the overall microbial communities in ST AS changed from an Actinobacteriota to a Proteobacteria-dominated community due to the addition of bleach solution [21]. This shift of microbial communities may explain the abrupt decrease of Saccharibacteria because bacteria within Actinobacteriota have been experimentally demonstrated as hosts for Saccharibacteria [7, 56]. The associations between Saccharibacteria and Actinobacteriota were also confirmed by the similarity-based network analysis. On the one hand, multiple network connections between given CPR bacterium and non-CPR bacteria with distinct taxonomic affiliations indicated the multiple hosts association of CPR bacteria. On the other hand, we should be noted that CPR bacteria within the same lineage (e.g., class) typically conserved a similar bacterial host distribution, suggesting the specificity of interactions between CPR and host bacteria. Together with the nature of limited metabolic potentials possessed by CPR bacteria in AS systems, we speculated that changes in CPR communities in AS systems were directly driven by the available bacterial hosts.

Saccharibacteria were widely identified in human oral and gut microbiomes [57]. It is expected that the human-associated Saccharibacteria should be identified and/or enriched in AS systems when considering the human wastes will be collected by sewage collection system and finally received by WWTPs. However, our analyses revealed that CPR bacteria in AS shared higher similarities of proteome content with environmental CRP bacteria than human-associated ones. Since microbial communities from different types of habitats are divergent from each other, this might to some degree explain the observed niche differentiation between CPR organisms in AS and the human body. Even for the same type of ecosystem, Christine et al. observed little shared CPR species across different groundwater sites [15]. These findings further confirmed that CPR community compositions and dynamics may be determined by the host populations. Comparative genomic analysis further provided gene signs of the adaptive response of CPR bacteria in AS systems. Genes for oxidative stress resistance were significantly enriched in AS CPR MAGs may enable CPR bacteria to adapt to the high oxygen condition in aeration tanks.

Despite the patchy metabolic capabilities of CPR bacteria, our results suggested these small, yet abundant bacteria might have involvements in the wastewater treatment process. The widely distributed polysaccharide lyase, glycoside hydrolase and carbohydrate-binding modules in CPR MAGs indicated that these bacteria might be involved in cooperative biogeochemical cycling, especially considering the ultrasmall cellular size may increase the surface area relative to cytoplasm volume of the bacterial host [52]. We also observed that Dojkabacteria, Gracilibacteria, Microgenomatia, and Saccharimonadia may act as fermenters in AS and contribute to the production of acetate, lactate, and formate, which might support the growth of CPR bacterial hosts [13]. These genetic findings collectively suggested that the exchange of metabolic productions between CPR bacteria and their hosts might not be just one way.

The observed horizontal gene transfer events between CPR bacteria and prokaryotic organisms or phages were evident for the impacts of CPR bacteria on microbial evolution. As reported in the experiments, saccharibacterium TM7x has a broad bacterial host range [56]. Therefore, CPR bacteria might be a key driver of bacterial evolution, with consideration of the broad range of CPR bacterial hosts. The enrichment of CPR bacteria in WWTPs effluent highlighted the importance of disinfection steps for conventional biological wastewater treatment systems, particularly considering the observed significant enrichment of tetracycline and multidrug resistance genes in WWTP-associated CPR bacteria. Unattached TM7x remains viable and could re-infect new bacterial hosts when available [58], suggesting the discharged CPR bacteria might contribute to the spread of ARGs in the wastewater effluent-receiving ecosystems.

## Conclusion

Overall, our longitudinal metagenomic analyses demonstrate the high bacterial diversities and abundances of CPR bacteria in AS system. The observed striking fluctuations in CPR communities and similarity-based networks collectively reveal that CPR bacteria might interact with multiple bacterial hosts with a specific taxonomic distribution. The limited metabolic and biosynthetic capabilities are also observed in the CPR guilds in AS systems, suggesting that the dynamics of CPR are directly driven by the available hosts. Although the studied WWTP was designed to treat municipal wastewater, proteome content similarities between human-associated and environmental CPR MAGs indicate that dominant CPR bacteria (Saccharibacteria) in AS systems may originate from environmental samples. Furthermore, the significantly enriched genes for oxygen stress resistance in AS CPR MAGs might enable them to become more adapted to high oxygen conditions compared to the human-associated ones, supporting the observed niche differentiation of CPR bacteria in AS system. Notably, our analyses highlight that CPR bacteria in AS might have involvements in carbohydrate hydrolysis and fermentation in wastewater treatment systems, as well as affect bacterial evolution via horizontal gene transfer. As the findings of this study are obtained based on in silico analyses, the real host range of CPR bacteria in AS systems and how they interact with their bacterial hosts are required to be answered in future investigations.

## Supplementary Information

**Additional file 1: Table S1.** Summary of 43 CPR markers used by CheckM for genome quality estimation. **Table S2.** Summary of the studied CPR MAGs in the present study. **Table S3.** Pairwise ANI analysis of all studied CPR MAGs in the present study. **Table S4.** Relative abundance of CPR bacteria in Shatin activated sludge samples. **Table S5.** Estimated completeness (%) of KEGG modules in the recovered CPR MAGs from Shatin activated sludge. **Table S6.** The relative abundances of the newly recovered bacterial MAGs in penicillin treated activated sludge metagenome. **Table S7.** Summary of public CPR bacteria used for pangenomic analysis. **Table S8.** Carbohydrate-active enzymes frequency in CPR bacteria recovered from activated sludge metagenomes of Shatin WWTP. **Table S9.** Summary of potential lacterial gene transfer events between CPR bacteria and other prokaryotic organisms (non-CPR bacteria and archaea) in Shatin WWTP. **Table S10.** Eggnog annotation results of the ORFs on putative horizontal transferred DNA fragments between CPR bacteria and prokaryotic organisms in Shatin WWTP. **Table S11.** Summary of potential horizontal gene transfer events between CPR bacteria and phages in Shatin WWTP. **Table S12**. Eggnog annotation results of the ORFs on putative horizontal transferred DNA fragments between CPR bacteria and phages in Shatin WWTP.

**Additional file 2:** **Figure S1.** Completeness estimation using different gene marker sets. **Figure S2.** Ridge plot shows the temporal dynamics of abundant saccharimonadial bacteria with relative abundance >0.5% in at least one activated sludge sample. The numbers in the ridge plot are the maximum relative abundance of CPR bacteria. The lowest taxonomic assignments of different CPR bacteria are shown in brackets. **Figure S3.** Ridge plot shows the temporal dynamics of abundant CPR bacteria from the same module and associated bacteria inferred by the microbial network. The lowest taxonomic assignments of different CPR bacteria are shown in brackets. **Figure S4.** Representation of CPR proteome content reporting how the pangenome varies as genomes are added in random order to the analysis. This analysis is conducted with pangenome of protein families with 5 or more members among CPR bacteria (a) and all protein families (b). **Figure S5.** COG categories of ORFs predicted by the putative lateral transferred DNA fragments. **Figure S6.** Relative abundance of CPR bacteria in activated sludge and effluent metagenomes generated from corresponding samples.

## Data Availability

The raw nucleotide sequence data used in the present study have been deposited in the NCBI database under project ID PRJNA432264.

## Abbreviations

ANI: Average nucleotide identity
AS: Activated sludge
ARG: Antibiotic resistance gene
CPR: Candidate phyla radiation
eLSA: Extended local similarity analysis
LS: Local similarity
MAGs: Metagenome-assembled genomes
MCL: Markov clustering algorithm
ORF: Open reading frame
ST: Shatin
SWH: Shek Wu Hui
STL: Stanley
WWTPs: Wastewater treatment plants
