# Supplementary material for: Genome-centric metagenomics reveals the host-driven dynamics and ecological role of CPR bacteria in an activated sludge system
Source: Microbiome. 2023 Mar 22;11:56. doi: 10.1186/s40168-023-01494-1 (PMC10031880; doi:10.1186/s40168-023-01494-1)
Supplement: Supplementary file 3 — Additional file 2: Figure S1. Completeness estimation using different gene marker sets. Figure S2. Ridge plot shows the temporal dynamics of abundant saccharimonadial bacteria with relative abundance >0.5% in at least one activated sludge sample. The numbers in the ridge plot are the maximum relative abundance of CPR bacteria. The lowest taxonomic assignments of different CPR bacteria are shown in brackets. Figure S3. Ridge plot shows the temporal dynamics of abundant CPR bacteria from the same module and associated bacteria inferred by the microbial network. The lowest taxonomic assignments of different CPR bacteria are shown in brackets. Figure S4. Representation of CPR proteome content reporting how the pangenome varies as genomes are added in random order to the analysis. This analysis is conducted with pangenome of protein families with 5 or more members among CPR bacteria (a) and all protein families (b). Figure S5. COG categories of ORFs predicted by the putative lateral transferred DNA fragments. Figure S6. Relative abundance of CPR bacteria in activated sludge and effluent metagenomes generated from corresponding samples. [file 40168_2023_1494_MOESM2_ESM.doc]

**Supplementary Information**

**for**

**Genome-centric metagenomics reveals the host-driven dynamics and ecological role of CPR bacteria in an activated sludge system**

Yulin Wang^1,2^, Yulin Zhang^2^, Yu Hu^1^, Lei Liu^2^, Shuang-Jiang Liu^1^, Tong Zhang^2^*

Yulin Wang: State Key Laboratory of Microbial Technology, Shandong University, Qingdao 266000, P. R. China; Environmental Microbiome Engineering and Biotechnology Laboratory, The University of Hong Kong, Hong Kong, China. [wangyulin@sdu.edu.cn](mailto:wangyulin@sdu.edu.cn).

Yulin Zhang: Environmental Microbiome Engineering and Biotechnology Laboratory, The University of Hong Kong, Hong Kong, China. [siazhang@connect.hku.hk](mailto:siazhang@connect.hku.hk).

Yu Hu: State Key Laboratory of Microbial Technology, Shandong University, Qingdao 266000, P. R. China. [202112582@mail.sdu.edu.cn](mailto:202112582@mail.sdu.edu.cn).

Lei Liu: Environmental Microbiome Engineering and Biotechnology Laboratory, The University of Hong Kong, Hong Kong, China. [liuleice@hku.hk](mailto:liuleice@hku.hk).

Shuangjiang Liu: State Key Laboratory of Microbial Technology, Shandong University, Qingdao 266000, P. R. China. [liusj@sdu.edu.cn](mailto:liusj@sdu.edu.cn).

Tong Zhang (Corresponding author): Environmental Microbiome Engineering and Biotechnology Laboratory, The University of Hong Kong, Hong Kong, China. [zhangt@hku.hk](mailto:zhangt@hku.hk).


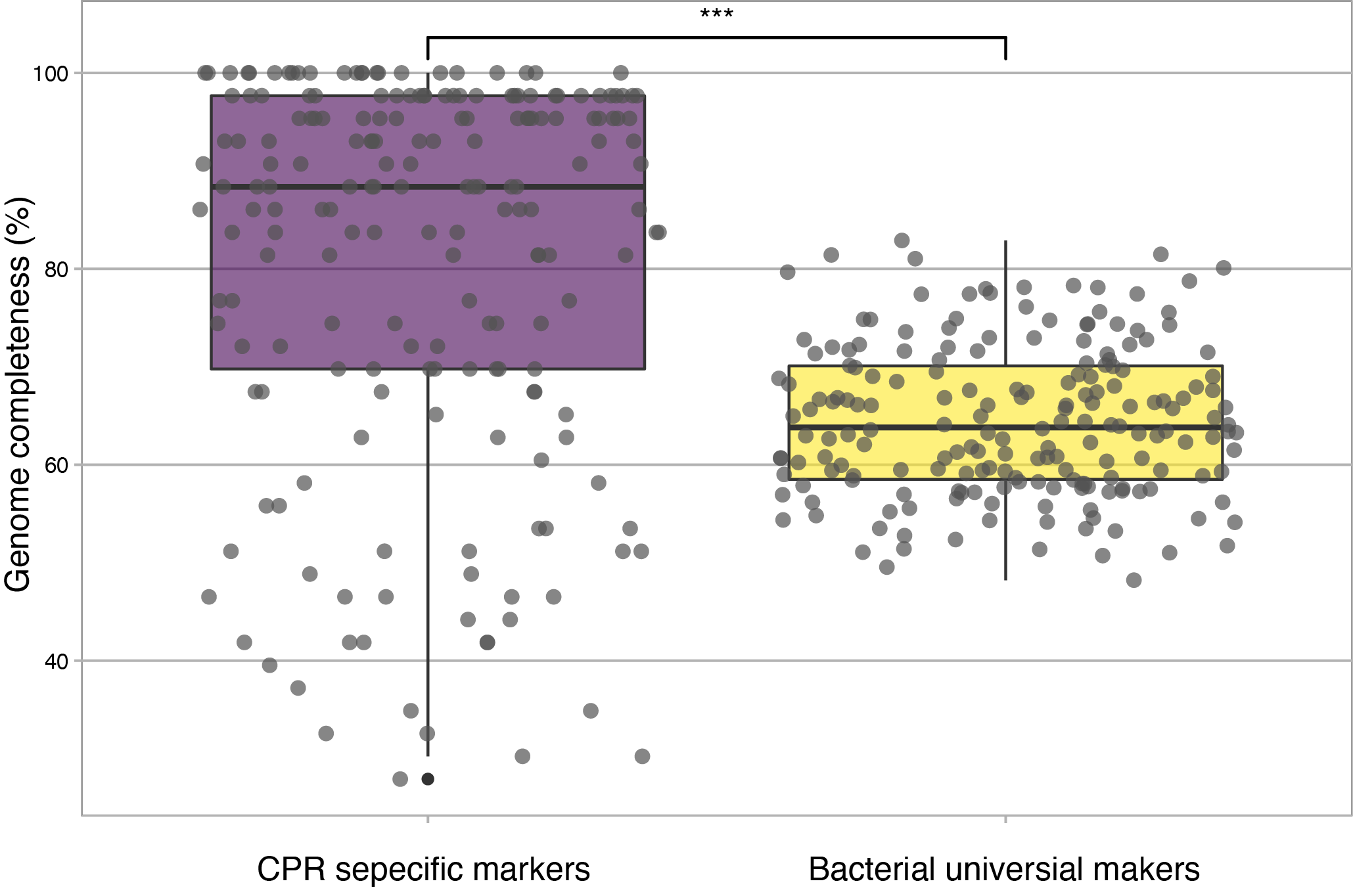


Figure S1. Completeness estimation using different gene marker sets.


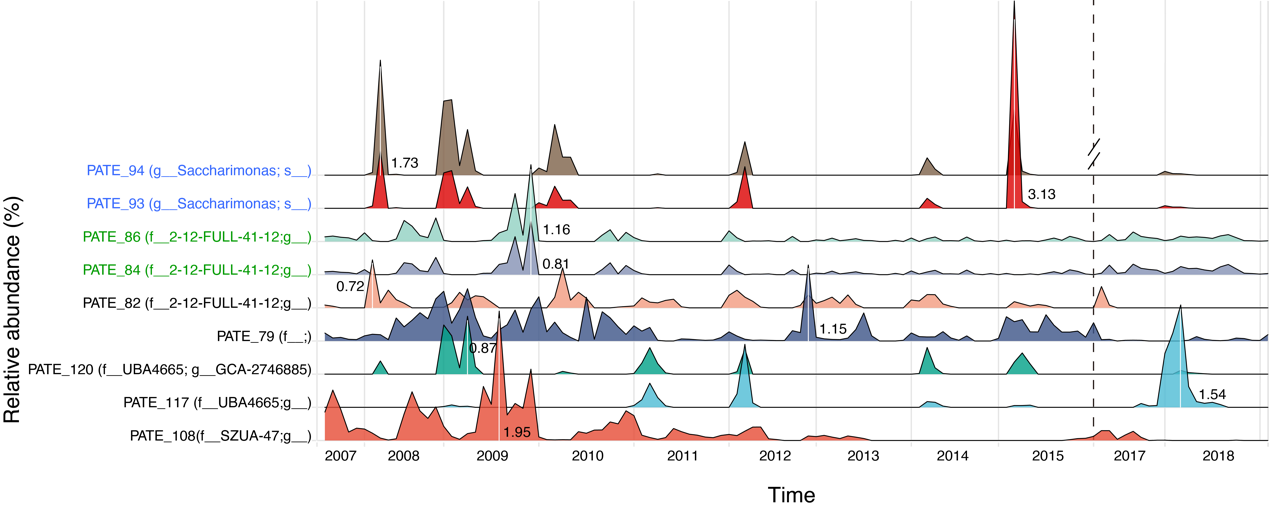


Figure S2. Ridge plot shows the temporal dynamics of abundant saccharimonadial bacteria with relative abundance >0.5% in at least one activated sludge sample. The numbers in the ridge plot are the maximum relative abundance of CPR bacteria. The lowest taxonomic assignments of different CPR bacteria are shown in brackets.


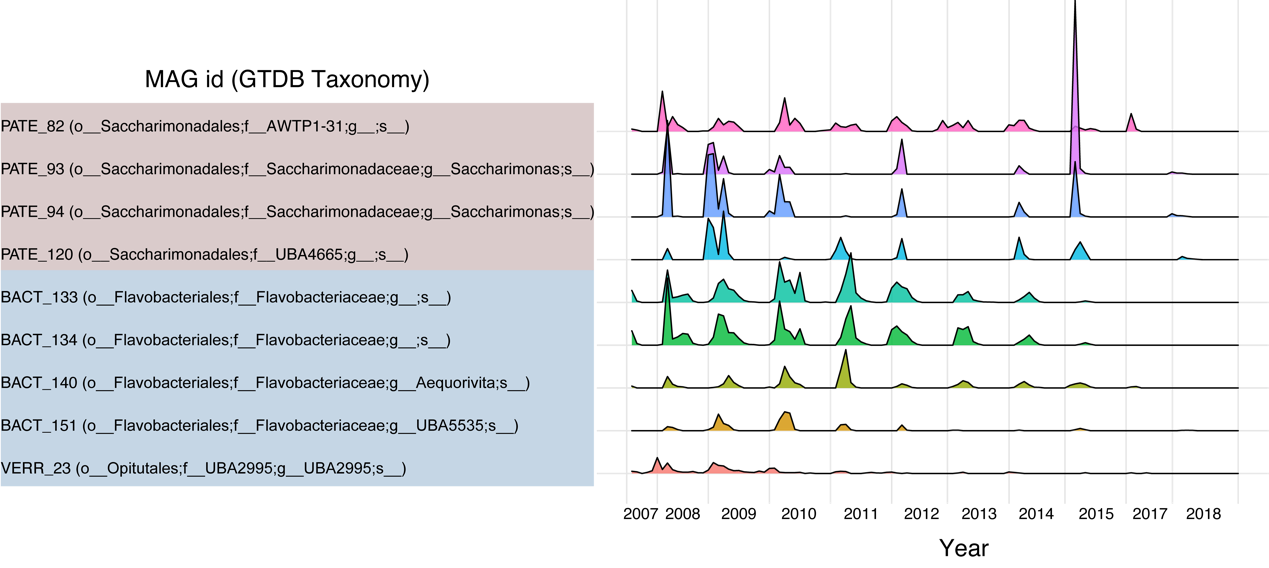


Figure S3. Ridge plot shows the temporal dynamics of abundant CPR bacteria from the same module and associated bacteria inferred by the microbial network. The lowest taxonomic assignments of different CPR bacteria are shown in brackets.


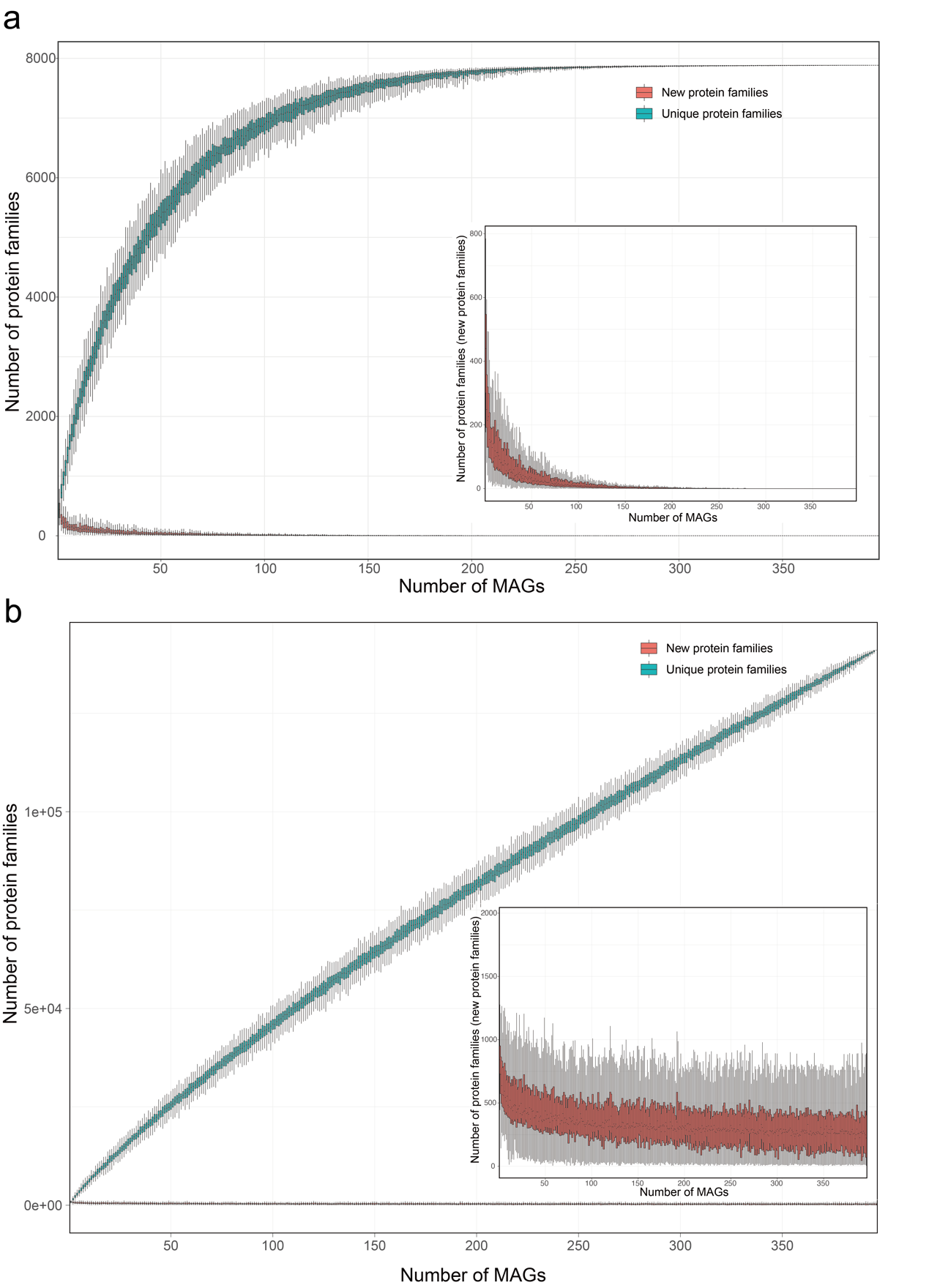


Figure S4. Representation of CPR proteome content reporting how the pangenome varies as genomes are added in random order to the analysis. This analysis is conducted with pangenome of protein families with 5 or more members among CPR bacteria (a) and all protein families (b).

Figure S5. COG categories of ORFs predicted by the putative lateral transferred DNA fragments.


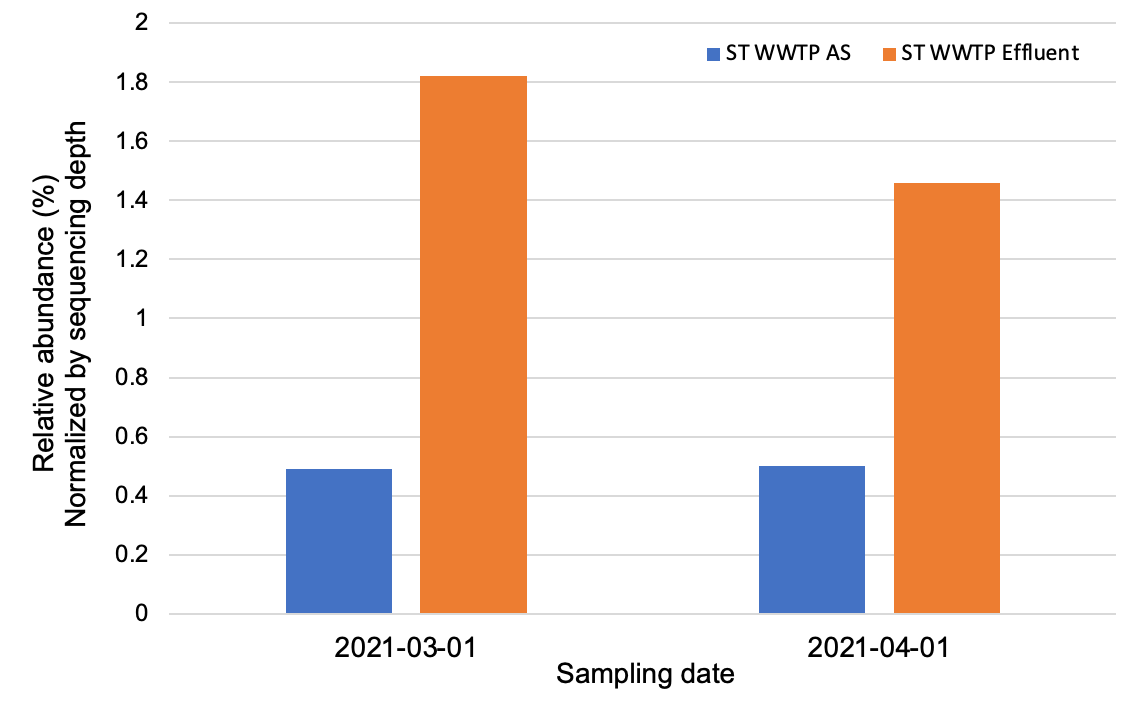


Figure S6. Relative abundance of CPR bacteria in activated sludge and effluent metagenomes generated from corresponding samples.
